# IL-37 Expression Reduces Lean Body Mass in Mice by Reducing Food Intake

**DOI:** 10.3390/ijms19082264

**Published:** 2018-08-02

**Authors:** Eline N. Kuipers, Andrea D. van Dam, Dov B. Ballak, Ellemiek A. de Wit, Charles A. Dinarello, Rinke Stienstra, Janna A. van Diepen, Patrick C.N. Rensen, Mariëtte R. Boon

**Affiliations:** 1 Department of Medicine, Division of Endocrinology, Leiden University Medical Center, Post Zone C7Q, P.O. Box 9600, 2300 RC Leiden, The Netherlands; e.n.kuipers@lumc.nl (E.N.K.); a.d.van_dam@lumc.nl (A.D.v.D.); ellemiekdewit@gmail.com (E.A.d.W.); p.c.n.rensen@lumc.nl (P.C.N.R.); 2 Einthoven Laboratory for Experimental Vascular Medicine, Leiden University Medical Center, P.O. Box 9600, 2300 RC Leiden, The Netherlands; 3 Department of Medicine, University of Colorado, Aurora, CO 80045, USA; dov.ballak@Radboudumc.nl (D.B.B.); cdinare333@aol.com (C.A.D.); 4 Department of General Internal Medicine and Radboud Center for Infectious Diseases, Radboud University Nijmegen Medical Center, 6525 HP Nijmegen, The Netherlands; rinke.stienstra@radboudumc.nl (R.S.); janna.vandiepen@radboudumc.nl (J.A.v.D.); 5 Nutrition, Metabolism and Genomics group, Division of Human Nutrition and Health, Wageningen University, 6703 HD Wageningen, The Netherlands

**Keywords:** IL-37, energy metabolism, food intake, high fat diet

## Abstract

The human cytokine interleukin (IL)-37 is an anti-inflammatory member of the IL-1 family of cytokines. Transgenic expression of IL-37 in mice protects them from diet-induced obesity and associated metabolic complications including dyslipidemia, inflammation and insulin resistance. The precise mechanism of action leading to these beneficial metabolic effects is not entirely known. Therefore, we aimed to assess in detail the effect of transgenic IL-37 expression on energy balance, including food intake and energy expenditure. Feeding homozygous IL-37 transgenic mice and wild-type (WT) control mice a high-fat diet (HFD; 45% kcal palm fat) for 6 weeks showed that IL-37 reduced body weight related to a marked decrease in food intake. Subsequent mechanistic studies in mice with heterozygous IL-37 expression versus WT littermates, fed the HFD for 18 weeks, confirmed that IL-37 reduces food intake, which led to a decrease in lean body mass, but did not reduce fat mass and plasma lipid levels or alterations in energy expenditure independent of lean body mass. Taken together, this suggests that IL-37 reduces lean body mass by reducing food intake.

## 1. Introduction

The worldwide prevalence of obesity, defined as a body mass index (BMI) >30 kg/m^2^, has nearly doubled since 1980, and at least 2.7 million people die each year as a result of obesity [1]. This number is expected to further increase over the next decade. Obesity has a great impact on public health as it leads to disorders such as dyslipidemia, type 2 diabetes and cardiovascular disease [2,3]. Obesity is caused by a positive energy balance that leads to excessive fat accumulation in adipose tissue, hypertrophy of adipocytes, hypoxia, and in the end cell death. This results in recruitment of inflammatory cells by white adipose tissue (WAT), which eventually leads to adipose tissue dysfunction, preceding dyslipidemia and type 2 diabetes [4,5].

It has been shown that release of pro-inflammatory cytokines by adipocytes and/or immune cells in adipose tissue, including IL-1β, IL-6, TNFα and CCL2, is higher during obesity compared to the lean state. More specifically, TNFα has been repeatedly shown to hamper insulin signaling and to result in insulin resistance, which may eventually lead to type 2 diabetes (reviewed in [6]). Inhibiting pro-inflammatory cytokines to counteract their disadvantageous metabolic effects is extensively being studied [7,8]. In contrast, anti-inflammatory cytokines that could potentially ameliorate the inflammatory microenvironment in obesity have been less well studied. IL-37, a cytokine previously known as IL-1 family member 7 (IL-1F7), is a member of the IL-1 family of cytokines which includes, amongst others, IL-1α, IL-1β, IL-1Ra, IL-18 and IL-33 [9]. The first studies into the function of IL-37 showed that it is a natural suppressor of innate inflammatory and immune responses [10]. In humans, several tissues and cell types express IL-37, including blood monocytes [11,12], epithelial cells [13], endothelial cells [14] and, importantly, also adipocytes [9,15]. IL-37 mRNA is degraded in the absence of inflammation, but upon lipopolysaccharide (LPS) stimulation, that is, a pro-inflammatory stimulus, IL-37 expression increases [16,17,18]. In vitro, it was shown that IL-37 suppresses expression of pro-inflammatory factors in monocytes and macrophages [10,17]. These studies indicate that IL-37 expression counteracts pro-inflammatory cytokine expression through controlled mechanisms that ensure balance between host defense functions of inflammation and adverse effects [10,16,17].

Since a mouse homolog of IL-37 is unknown, in vivo studies into IL-37 function have employed a mouse model with transgenic (tg) expression of the most abundant isoform of human IL-37 (IL-37b; IL-37tg mice) [10,11,15,19,20,21]. IL-37tg mice are protected from LPS-induced shock related to lower levels of inflammatory cytokines upon LPS treatment [10]. Strikingly, IL-37 expression in mice also protects against obesity and obesity-associated inflammation and insulin resistance [15]. IL-37tg mice fed a high-fat diet (HFD) showed a markedly lower gain in body weight and lower plasma lipid levels compared to wild-type (WT) control mice [15]. Although IL-37 was shown to induce metabolic changes in adipocytes and immune cells [15,22,23], it is still not entirely known how the beneficial metabolic effects are instigated. Therefore, the aim of this study was to investigate the effect of IL-37 on energy balance, that is, energy intake and energy expenditure, in more detail. To this end, IL-37tg and wild-type (WT) mice were fed a high-fat diet (HFD) to induce obesity. Homozygous IL-37tg mice showed lower body weight and food intake. Mechanistic studies in heterozygous mice demonstrated that IL-37 lowers food intake and specifically reduces lean body mass without reducing fat mass or plasma lipid levels. These results suggest that the lower lean body mass in IL-37tg mice is a result of lower food intake.

## 2. Results

### 2.1. IL-37 Expression Alleviates Diet-Induced Weight Gain and Reduces Food Intake

**Figure 1:**
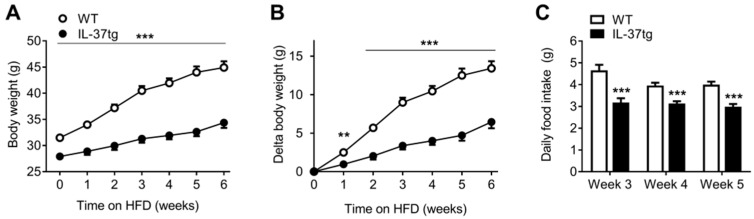
IL-37 expression alleviates diet-induced weight gain and reduces food intake. 10-week old male C57Bl/6J mice and homozygous IL-37tg mice on a C57Bl/6J background were fed a high-fat diet (HFD) for 6 weeks. Body weight (**A**,**B**) and food intake (**C**) were measured at indicated time points. Values represent means ± SEM (*n* = 10 animals per group). ** *p* < 0.01, *** *p* < 0.001 vs. wild-type (WT).

To assess the effect of IL-37 expression on the development of HFD-induced obesity, homozygous IL-37tg mice were fed a HFD for 6 weeks. Body weight of the IL-37tg mice was already 3.6 g lower compared to the WT control mice before initiation of the HFD (Figure 1A) and this difference was magnified during HFD feeding resulting in a body weight difference of 10.5 g after 6 weeks of HFD (*p* < 0.001). Even when corrected for body weight at the start of HFD feeding, IL-37tg mice gained substantially less weight compared to WT mice during 6 weeks of HFD (Figure 1B). Next, we measured whether the energy intake in IL-37tg mice was different compared to WT controls. Quite surprisingly, food intake in IL-37tg mice was markedly lower throughout the HFD intervention period (−21 to −32%, *p* < 0.001, Figure 1C). This suggests that IL-37tg expression might alleviate diet-induced weight gain by reducing food intake.

### 2.2. Reduced Food Intake in Heterozygous IL-37tg Mice Decreases Lean Body Mass

**Figure 2:**
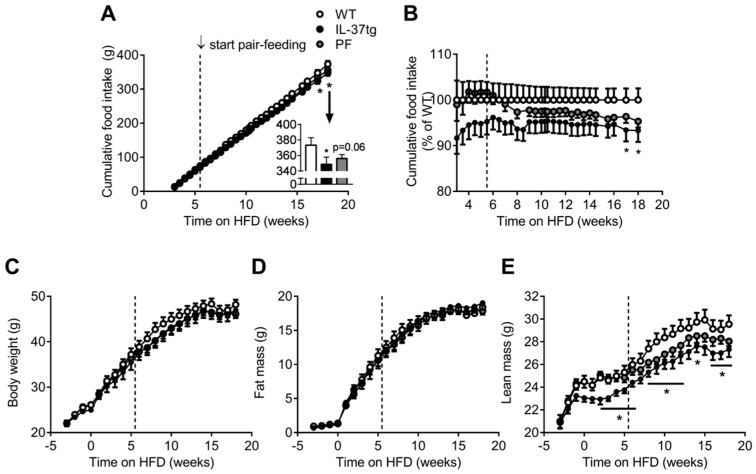
Reduced food intake in heterozygous IL-37tg mice decreases lean body mass. 10-week old male C57Bl/6J mice and heterozygous IL-37tg mice on a C57Bl/6J background were fed a high-fat diet (HFD) for 18 weeks. Pair-feeding of wild-type (WT) mice (PF group) was initiated after 5.5 weeks of HFD feeding and is indicated by the dotted line (**A**–**D**). Food intake (FI; **A**,**B**), body weight (**C**), fat mass (**D**) and lean mass (**E**) were measured at indicated time points. Values represent means ± SEM (*n* = 10 animals per group). * *p* < 0.05 IL-37tg vs. wild-type (WT).

**Figure 3:**
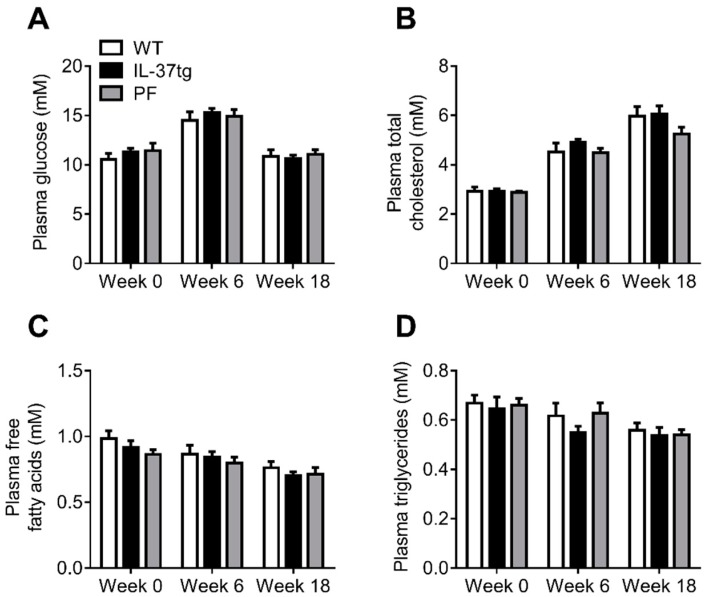
Heterozygous IL-37 expression does not affect plasma glucose and lipid levels. 10-week old male C57Bl/6J mice and heterozygous IL-37tg mice on a C57Bl/6J background were fed a high-fat diet (HFD) for 18 weeks. Pair-feeding of wild-type (WT) mice (PF group) was initiated after 5.5 weeks of HFD feeding. Plasma glucose (**A**), total cholesterol (**B**), free fatty acids (**C**) and triglycerides (**D**) were measured at indicated time points. Values represent means ± SEM (*n* = 10 animals per group).

To further investigate the effect of the reduced food intake in IL-37tg mice on body weight and composition independent of genetic background, a second study was set up that included a group of HFD-fed heterozygous IL-37tg mice and WT littermates that was pair-fed to these IL-37tg mice. Although less pronounced, heterozygous IL-37tg mice also had reduced food intake compared to WT littermates (−8% cumulative food intake after 18 weeks, *p* < 0.05, Figure 2A,B). Compared to homozygous IL-37tg mice, heterozygous IL-37tg mice only showed a tendency towards reduced body weight (Figure 2C). Estimation of body composition by EchoMRI revealed that, while heterozygous IL-37tg mice had similar fat mass compared to WT littermates (Figure 2D), their lean body mass was reduced (−7% after 18 weeks of HFD, *p* < 0.05). Remarkably, lean body mass of the pair-fed group aligned with the WT control group until pair-feeding was started, upon which it dropped towards the lean body mass of the IL-37tg mice (Figure 2E), suggesting that the reduction in food intake underlies the reduced lean body mass. To determine whether heterozygous IL-37 expression affects plasma glucose and lipid levels, blood was drawn after 0, 6 and 18 weeks of HFD feeding. Plasma glucose, total cholesterol, free fatty acids and triglyceride levels did not differ between the groups (Figure 3A–D). These data suggest that heterozygous IL-37 overexpression primarily affects lean body mass via reducing food intake without affecting plasma glucose and lipid levels.

### 2.3. IL-37 Expression Decreases Energy Expenditure in Conjunction with Lean Body Mass Reduction

**Figure 4:**
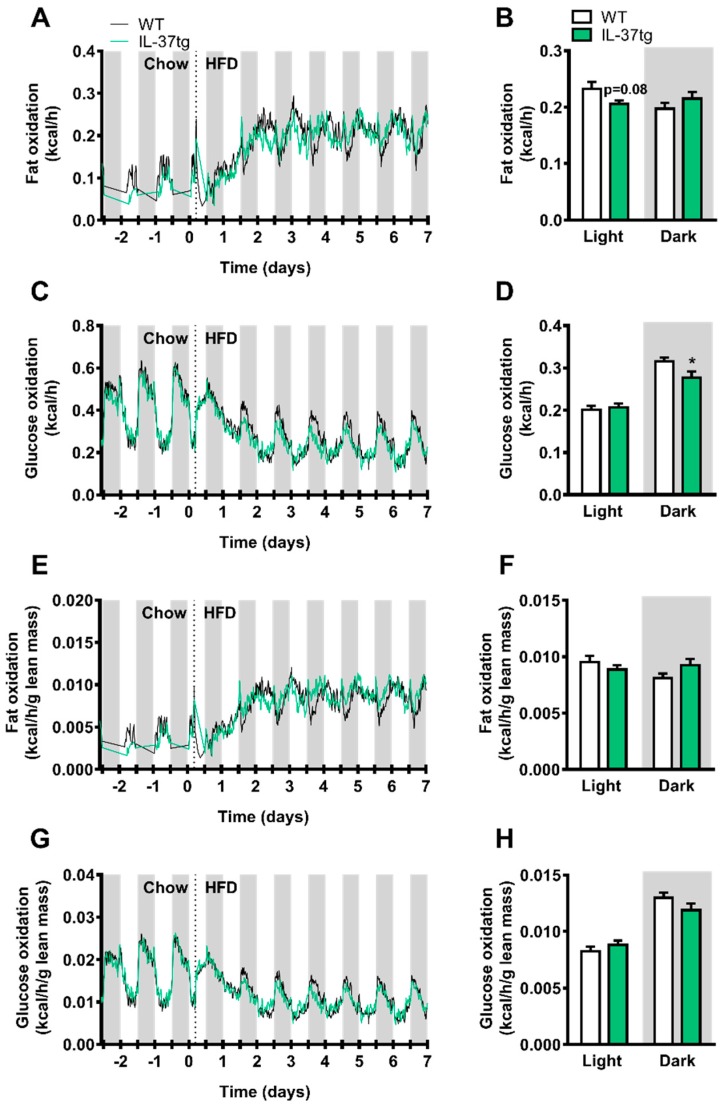
Heterozygous IL-37 expression decreases glucose oxidation in conjunction with lean body mass reduction. 10-week old male C57Bl/6J mice and heterozygous IL-37tg mice on a C57Bl/6J background were fed a high-fat diet (HFD). From 2 days before initiation of HFD until 1 week after the switch to HFD, mice were housed in fully automatic metabolic cages, which measured oxygen uptake (V_O_2__) and carbon dioxide production (V_CO_2__). Fat (**A**,**B**) and glucose (**C**,**D**) oxidation was calculated and were corrected for lean mass (fat oxidation; **E**,**F**; glucose oxidation; **G**,**H**). Bar graphs were based on calculations of the mean from day 1.5 to 7. Values represent means (**A**,**C**,**E**,**G**) and bar graphs represent means ± SEM (**B**,**D**,**F**,**H**) (*n* = 8 animals per group). * *p* < 0.05 IL-37tg vs. wild-type (WT).

To assess the effect of IL-37 expression on energy metabolism in more depth, energy expenditure, substrate utilization and activity levels were monitored by metabolic cages two days before and during the first week of HFD feeding. IL-37 expression did not affect physical activity levels (Figure S1A,B). Fat oxidation during the light period tended to be lower in IL-37tg mice compared to WT littermate controls (−12%, *p* = 0.08, Figure 4A,B). Glucose oxidation during the dark period, in which mice are active, was lower in the IL-37tg mice compared to the control group (−12%, *p* < 0.05, Figure 4C,D). Energy expenditure during the light period tended to be lower in IL-37tg mice compared to WT littermate controls (−5%, *p* = 0.07, Figure S1C,D). Since IL-37 expression reduced lean body mass, which is an important contributor to energy expenditure, we corrected the fat oxidation, glucose oxidation and total energy expenditure for lean body mass and found that differences in substrate utilization and total energy expenditure lost significance between IL-37tg and WT control mice (Figure 4E–H, Figure S1E,F). These data suggest that heterozygous overexpression of IL-37 decreases glucose oxidation and tends to decrease fat oxidation and energy expenditure as a consequence of reduced lean body mass.

## 3. Discussion

IL-37 is an anti-inflammatory cytokine of the immune system, and transgenic expression of IL-37 in mice protects them from diet-induced obesity and associated metabolic complications including dyslipidemia, inflammation and insulin resistance [15]. In the current study, we investigated the effect of transgenic IL-37 expression on energy balance in more detail. We confirmed that mice homozygously expressing IL-37 had lower body weight upon HFD feeding, and went on to show that these animals had a marked decrease in food intake. Subsequent mechanistic studies in mice with heterozygous expression showed that IL-37 reduces food intake which led to a decrease in lean body mass, but did not reduce fat mass and plasma lipid levels or alterations in energy expenditure independent of lean body mass. Taken together, this indicates that IL-37 expression lowers lean body mass at least partly via reducing food intake.

We found that mice homozygously expressing IL-37 were leaner than WT control mice already before onset of the HFD, and this difference became more evident during HFD feeding for 6 weeks. These body weight curves differ from the course of body weight found previously by Ballak et al., [15] for which the exact explanation is currently unknown to us. Despite the fact that the diet, age of the animals and the experimental facility were the same in both studies, IL-37tg and WT control mice in the previous study had a similar weight before onset of the HFD and lower body weight in IL-37tg mice was not evident until 6 weeks of HFD feeding [15]. Strikingly, the previous study reported similar food intake between IL-37tg and WT control mice. The difference in results between our results and the previous experiments published by Ballak et al. [15] may be due to differences in methods to monitor food intake. In addition, non-littermates were used in the first experiment in this paper and in the study by Ballak et al. [12]. Therefore, the reduction in fat mass and metabolic phenotype that were found initially could also be due to differences in genetic make-up or composition of gut microbiota [24,25].

For subsequent mechanistic studies into the beneficial metabolic phenotype observed in IL-37tg mice independent of genetic makeup we used heterozygous IL-37tg and WT littermate control animals. The reason we switched to a heterozygous mouse model was because a breeding that generated WT littermate controls was needed and it was impossible to distinguish homozygous from heterozygous mice by PCR, a heterozygous breeding was kept in which heterozygous IL-37tg mice were crossed with WT mice. We observed no obvious difference in behavior between transgenic and WT animals as described before [10]. Although we attempted to investigate whether reduced food intake in IL-37tg animals was causal to the beneficial metabolic phenotype by performing a study in which we included a pair-fed group, we could not reproduce the marked effect on total body weight that we found initially and that was also reported previously [15]. A downside of using this approach is that expression of IL-37 in heterozygous IL-37tg mice will be lower than in homozygous IL-37tg mice, explaining the reduced effect observed on body weight. Indeed, in heterozygous IL-37tg mice the *IL-37* mRNA expression has shown to be lower and the concomitant beneficial effects milder than the expression and effects of homozygous IL-37tg mice [10,26]. Since there seems to be a gene dose-dependent effect of IL-37 on food intake and total body weight, and heterozygous IL-37 expression lowers specifically lean body mass, it might be that homozygous IL-37 expression lowers body weight by reducing both fat and lean body mass. Unfortunately, we did not take measures of body composition of the homozygous IL-37tg mice to confirm this. To circumvent the use of transgenic mouse models, an alternative approach for future studies on IL-37 function would be the use of recombinant IL-37 [27].

IL-37 production has been reported to be low and it is mainly induced and detected upon pro-inflammatory stimuli, such as LPS [10] or others that would accumulate during the development of obesity. Even though the animals in our study were fed a pro-inflammatory diet containing palm oil, which may have led to higher LPS exposure in our models [28], heterozygous IL-37 expression might not have been sufficient to counterbalance inflammation upon the pro-inflammatory diet. Nevertheless, heterozygous IL-37tg mice still had a reduction in food intake and less lean body mass. Restricting energy intake in mice with roughly 26%, which is three times higher than the energy restriction in our study, has been shown to decrease lean body mass by about 11% (compared to −7% in our study) without affecting fat mass before [29]. Since in our study the pair-fed mice also showed a reduction in lean body mass from the moment the pair-feeding was initiated, lower lean body mass in IL-37tg mice is probably a result of lower food intake.

How IL-37 might reduce food intake is as yet unknown and would be an interesting subject of future investigation. Extracellular IL-37 interacts with IL-1R8 (SIGIRR) and IL-18Rα. The latter is also the receptor of the pro-inflammatory cytokine IL-18, which is a member of the IL-1 family of cytokines as well [22]. Interestingly, IL-18 deficiency leads to increased food intake and body weight. In addition, intracerebral administration of rIL-18 reduces food intake, suggesting that IL-18 acts centrally [30]. Very recently, Francesconi et al. [31] discovered that the IL-18 receptor is highly expressed in the bed nucleus of the stria terminalis, a part of the extended amygdala that is known to influence feeding by projecting on the lateral hypothalamus. It is therefore possible that IL-37 inhibits food intake by acting on the IL-18 receptor in the brain. Furthermore, we cannot exclude the possibility that IL-37 modulates the expression of other cytokines and thereby influences food intake.

Whether IL-37 affects food intake in humans, who naturally express IL-37, is unknown. IL-37 gene expression in adipose tissue of humans was found to be higher in subjects with low adipose tissue leptin protein levels [15], which could suggest that high IL-37 expression lowers food intake and consequently reduces levels of the satiety hormone leptin in adipose tissue. Elevated IL-37 levels in humans are generally found in patients with inflammatory diseases such as nonallergic asthma [32] and systemic lupus erythematosus (SLE) [33]. SLE patients have indeed been reported to have inadequate food intake [34], which would be consistent with higher IL-37, but the complexity and versatility of the immune system makes it challenging to attribute this effect to a specific inflammatory component. Recent genetic studies on body mass index revealed that a majority of the tissues and cell types in which genes near BMI-associated SNPs are highly expressed, are part of the central nervous system [35]. This provides strong support for an important role of the central nervous system, which contains the key sites of central appetite regulation, in obesity susceptibility and therefore highlights the importance of research into factors that influence appetite, such as IL-37. Future studies for therapeutics in humans will focus on recombinant IL-37 linked to the Fc domain of mouse IgG1 (fusion protein), in order to increase the in vivo efficacy of IL-37 on inflammatory and immune-mediated diseases.

In conclusion, IL-37 expression in mice reduces food intake, which may underlie the beneficial metabolic effects including fat mass reduction that have previously been reported in IL-37 transgenic mice. The mechanisms behind these findings and the pathophysiological significance of these findings in obesity in humans remain to be determined.

## 4. Materials and Methods

### 4.1. Animals and Diet

For the first study, male C57Bl/6J mice and IL-37tg mice homozygously overexpressing human IL-37 on a C57Bl/6J background were used. IL-37tg mice were generated as previously described [10], and C57Bl/6J mice were purchased from Jackson Laboratories (Bar Harbor, ME, USA). For the second study, male IL-37tg mice heterozygously expressing human IL-37 and WT littermates (both C57Bl/6J background) were bred. In both studies, mice were individually housed under standard conditions with a 12:12 h light-dark cycle and access to food and water. Animals were approximately 10 weeks of age at the start of the high-fat diet (HFD; D12451, of which lard fat was replaced by palm fat, purchased from Ssniff, Soest, The Netherlands and Research Diet Services, Wijk bij Duurstede, The Netherlands) feeding. The HFD contained 45% kcal from palm fat, 20% of kcal derived from protein and 35% kcal derived from carbohydrates, and was given for 6 or 18 weeks, as indicated. Mouse experiments were performed in accordance with the Institute for Laboratory Animal Research Guide for the Care and Use of Laboratory Animals and had received approval from the University Ethical Review Boards (DEC 13159, 2 September 2013, Radboud University Medical Centre, Nijmegen and Leiden University Medical Centre, The Netherlands).

### 4.2. Body Weight, Body Composition and Food Intake

At indicated time points, body weight and food intake were measured. Body composition was measured using an EchoMRI-100 analyser (EchoMRI, Houston, TX, USA). Because IL-37 expression induced hypophagia in the first experiment with IL-37tg homozygous mice, in the second experiment an additional group of C57Bl/6J mice that was pair-fed to the heterozygous IL-37tg group was taken along. To achieve pair-feeding, food intake of the ad libitum-fed IL-37tg mice was monitored daily and the pair-fed mice were given the average amount of diet that the IL-37tg mice had consumed the previous day, at the end of the light phase.

### 4.3. Plasma Glucose and Lipids

At the indicated time points, 6 h-fasted blood samples were collected by tail vein bleeding into chilled capillaries that were coated with paraoxon (Sigma-Aldrich, St. Louis, MO, USA) to prevent ongoing lipolysis [36]. Isolated plasma was assayed for glucose (Instruchemie, Delfzijl, The Netherlands), total cholesterol and triglycerides (Roche Diagnostics, Mannheim, Germany), and free fatty acids (Wako Diagnostics; Instruchemie, Delfzijl, The Netherlands), following the manufacturers’ protocols.

### 4.4. Energy Metabolism

Heterozygous IL-37tg mice and WT littermates were housed in fully automatic metabolic cages (LabMaster System; TSE Systems, Bad Homburg, Germany) several days before HFD feeding and the first week of HFD-feeding, as indicated. Metabolic cages measured oxygen uptake (V_O_2__) and carbon dioxide production (V_CO_2__). Glucose oxidation and fat oxidation were calculated from V_O_2__ and V_CO_2__ as described previously [37]. Total energy expenditure was calculated from V_O_2__ and V_CO_2__ using the Weir equation [38]. Physical activity was measured with infrared sensor frames.

### 4.5. Statistical Analysis

All data are expressed as means ± SEM. Differences between groups were determined using a two-tailed unpaired Student’s *t*-test and food intake measurements in the heterozygous IL-37tg mice using a one-tailed unpaired Student’s *t*-test. Statistical analyses were performed using Excel or SPSS 20.0 software package for Windows. Probability values less than 0.05 were considered statistically significant.

## Supplementary Materials

Supplementary materials can be found at http://www.mdpi.com/1422-0067/19/8/2264/s1.

## Abbreviations

BP	Binding protein
HFD	High fat diet
IL	Interleukin
SLE	Systemic lupus erythematosus
tg	Transgenic
WAT	White adipose tissue
WT	Wild-type
