# Supplementary material for: IL-37 Expression Reduces Lean Body Mass in Mice by Reducing Food Intake
Source: Int J Mol Sci. 2018 Aug 2;19(8):2264. doi: 10.3390/ijms19082264 (PMC6121375; doi:10.3390/ijms19082264)
Supplement: Supplementary file 1 [file ijms-19-02264-s001.pdf]

Supplementary data

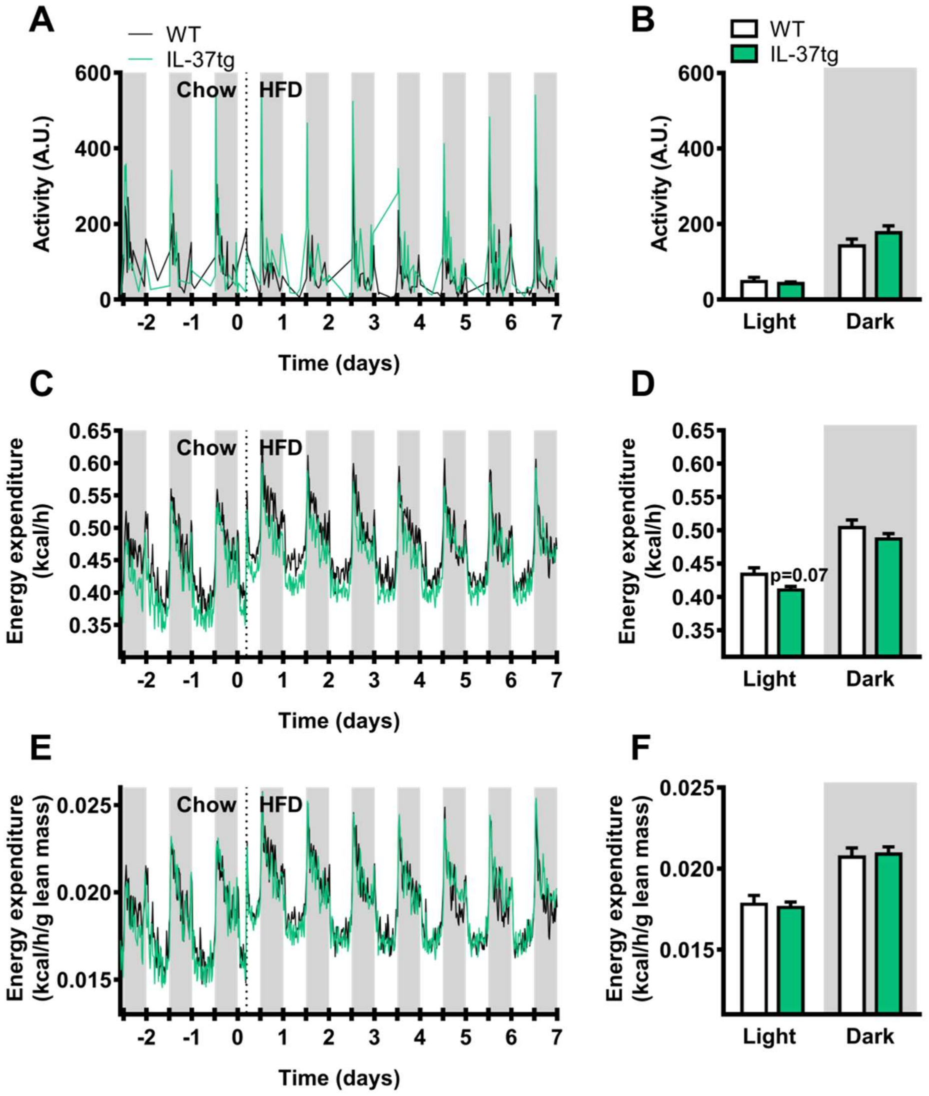

Figure S1. Heterozygous IL-37 expression decreases energy expenditure in conjunction with lean mass reduction. 10-week old male C57Bl/6J mice and heterozygous IL-37tg mice on a C57Bl/6J background were fed a high-fat diet (HFD). From 2 days before initiation of HFD until 1 week after the switch to HFD, mice were housed in fully automatic metabolic cages, which measured oxygen uptake (V_{O2}) and carbon dioxide production (V_{CO2}). Physical activity (**A**,**B**) was measured with infrared sensor frames. Total energy expenditure (**C**,**D**) and the energy expenditure were corrected for lean mass (**E**,**F**) was calculated from V_{O2} and V_{CO2} using the Weir equation. Bar graphs were based on calculations of the mean from day 1.5 to 7. Values represent means (**A**,**C**,**E**) and bar graphs represent means ± SEM (**B**,**D**,**F**) (n = 8 animals per group).
